# *Leishmania (L.) amazonensis* LaLRR17 increases parasite entry in macrophage by a mechanism dependent on GRP78

**DOI:** 10.1017/S0031182023000720

**Published:** 2023-09

**Authors:** Mauricio S. Peña, Fenny Hui Fen Tang, Fernando Alves de Lima Franco, Alessandro Taunay Rodrigues, Guilherme Moreira Paiva Carrara, Thaís Larissa Silva Araujo, Ricardo José Giordano, Giuseppe Palmisano, Maristela Martins de Camargo, Silvia Reni Bortolin Uliana, Beatriz Simonsen Stolf

**Affiliations:** 1 Department of Parasitology, Institute of Biomedical Sciences, University of São Paulo, São Paulo, Brazil; 2 Department of Biochemistry, Institute of Chemistry, University of São Paulo, São Paulo, Brazil; 3 Department of Immunology, Institute of Biomedical Sciences, University of São Paulo, São Paulo, Brazil

**Keywords:** GRP78, infection, LaLRR17, *Leishmania (L.) amazonensis*, macrophage, phagocytosis

## Abstract

Leishmaniases affect 12 million people worldwide. They are caused by *Leishmania* spp., protozoan parasites transmitted to mammals by female phlebotomine flies. During the life cycle, promastigote forms of the parasite live in the gut of infected sandflies and convert into amastigotes inside the vertebrate macrophages. The parasite evades macrophage's microbicidal responses due to virulence factors that affect parasite phagocytosis, survival and/or proliferation. The interaction between *Leishmania* and macrophage molecules is essential to phagocytosis and parasite survival. Proteins containing leucine-rich repeats (LRRs) are common in several organisms, and these motifs are usually involved in protein–protein interactions. We have identified the LRR17 gene, which encodes a protein with 6 LRR domains, in the genomes of several *Leishmania* species. We show here that promastigotes of *Leishmania (L.) amazonensis* overexpressing LaLRR17 are more infective *in vitro*. We produced recombinant LaLRR17 protein and identified macrophage 78 kDa glucose-regulated protein (GRP78) as a ligand for LaLRR17 employing affinity chromatography followed by mass spectrometry. We showed that GRP78 binds to LaLRR17 and that its blocking precludes the increase of infection conferred by LaLRR17. Our results are the first to report LRR17 gene and protein, and we hope they stimulate further studies on how this protein increases phagocytosis of *Leishmania*.

## Graphical abstract

**Figure:**
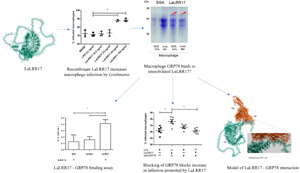

## Introduction

Leishmaniases are diseases caused by trypanosomatids of the *Leishmania* genus, with diverse clinical presentations ranging from self-healing cutaneous ulcers to a visceral form, potentially fatal if untreated (Reithinger *et al*., 2007). Approximately 12 million people are presently estimated to be suffering from this protozoal disease in Africa, Asia, America and Europe (Alvar *et al*., 2013), and about 20 different species of *Leishmania* may cause leishmaniasis in humans (Akhoundi *et al*., 2016). The clinical form of the disease depends on parasite species and on host immune response (McMahon-Pratt and Alexander, 2004).

The parasite's life cycle involves the extracellular promastigote stage in the intestine of the phlebotomine sand fly vector, and the intracellular amastigote in the vertebrate host (Bates, 2007). Promastigotes transmitted by the vector are phagocytosed by different cells but reside and multiply mainly in parasitophorous vacuoles of macrophages (Naderer and McConville, 2011). Both stages of the parasite are phagocytosed after adhesion to different macrophage receptors, which are crucial in determining the parasite's intracellular fate (Ueno and Wilson, 2012). The parasite intake process also involves components of the endoplasmic reticulum (ER) (Gagnon *et al*., 2002). Indeed, *Leishmania* parasitophorous vacuoles interact continuously with ER compartments and can recruit components that are important for the intracellular survival of the parasite (Ndjamen *et al*., 2010), and inhibition of this fusion leads to a reduction of the infection (Canton *et al*., 2012; Dias-Teixeira *et al*., 2016). In fact, a proteome analysis identified several ER-resident proteins in phagosomes such as calreticulin, calnexin, 78 kDa glucose-regulated protein (GRP78), endoplasmin and protein disulfide (PDI) (Garin *et al*., 2001), suggesting that components of the ER may fuse with the macrophage plasma membrane in the process known as ER-mediated phagocytosis (Desjardins, 2003; Canton *et al*., 2012; Dias-Teixeira *et al*., 2016).

The parasite uses multiple strategies to overcome the innate and acquired immune defence mechanisms and survive inside the macrophage (Lambertz *et al*., 2012; Bifeld and Clos, 2015). For example, the interaction of *Leishmania* surface molecules and macrophages leads to an inhibition of reactive oxygen species production and a decrease in the production of pro-inflammatory cytokines (Lambertz *et al*., 2012; Bifeld and Clos, 2015). The subversion of the host cell response is most likely dependent on parasite's products that find access to the host cell and interact with macrophage proteins or receptors, disrupting signalling pathways (Podinovskaia and Descoteaux, 2015). While it is known that *Leishmania* secretes a diverse repertoire of molecules, few of them have been functionally characterized. Lipophosphoglycan and GP63 are the most studied virulence factors in *Leishmania* (Isnard *et al*., 2012; Podinovskaia and Descoteaux, 2015). Many virulence factors such as GP63, secreted acid phosphatase, heat shock proteins (HSPs 70, 90 and 100) and tryparedoxin peroxidase are secreted by the parasite (Silverman *et al*., 2010; Lambertz *et al*., 2012).

Part of *Leishmania* virulence factors is stage regulated. In a search for virulence factors, our group identified 2 genes in chromosome 17, named *META1* and *META2*, which were more expressed in metacyclic than procyclic promastigotes (Uliana *et al*., 1999; Ramos *et al*., 2011). While studying these genes, we identified an open reading frame (ORF) near the *Leishmania (L.) amazonensis META1* gene that encodes a protein containing 6 leucine-rich repeat (LRR), which was named LaLRR17.

LRR is one of the most common protein motifs involved in protein–protein interactions (Kobe and Kajava, 2001). The repeat is composed of 20–30 amino acids with an 11-residue core element; several copies in tandem lead to a horseshoe structure that is believed to be at the source of the interactive properties of these proteins (Kobe and Kajava, 2001). The LRR family includes thousands of proteins, found from mammals to plants, taking part in interactions of various orders, including between parasites and host cells (Kobe and Kajava, 2001; Kedzierski *et al*., 2004*b*). In fact, LRRs are present in extracellular regions of Toll-like receptors and in intracellular nucleotide-binding and oligomerization domain (NOD) receptors (Girardin *et al*., 2002; McGuinness *et al*., 2003), which are important for pathogen recognition. LRRs also play important roles in *Listeria monocytogenes* infection. Internalins A and B are surface proteins necessary for *Listeria* internalization by several cells, and internalin LRRs and inter repeat regions were shown to be necessary and sufficient for host cell invasion (Lecuit *et al*., 1997).

In various *Leishmania* genomes already characterized there are 70–80 LRR-containing protein sequences. These include genes encoding surface molecules such as parasite surface antigen (PSA), gp46, various proteophosphoglycans (ppg3, ppg4, ppg5) and several putative proteins. Two proteins, proteophosphoglycan (PPG) and PSA-2, are important molecules involved in binding and phagocytosis of *Leishmania* by the macrophage (Kedzierski *et al*., 2004*a*). PSA-2 was detected in the extracellular region of the promastigote glycocalyx and contains 13 LRRs that interact with macrophage receptors (Kedzierski *et al*., 2004*b*). A protein identified in *Leishmania (L.) donovani* similar to the Internalin-A-like (Inl-A) from *L. monocytogenes* contains an LRR region that interacts with E-cadherin present in the host cell membrane (Mukherjee *et al*., 2016).

Here we report the characterization of the LRR17 gene and its expressed product. We also show that LaLRR17 increases adhesion of the parasite to the macrophage and *in vitro* infection by *L. (L.) amazonensis*. This effect is dependent on the binding to GRP78 in the macrophage surface. This is, to our knowledge, the first description of GRP78 involvement in *Leishmania* infection.

## Materials and methods

### *Leishmania (L.) amazonensis* promastigotes

Promastigotes of *L*. (*L*.) *amazonensis* from the MHOM/BR/73/M2269 strain were cultured at 24°C in M199 medium supplemented with 10% fetal calf serum (FCS). Transgenic *L. (L.) amazonensis* (pXG1 NEO LaLRR17::myc/His) and (pXG1 NEO) lines were cultured under the same conditions but in the presence of 150 μg mL^−1^ G418. Parasites were subcultured every 7 days to inoculums of 2 × 10^6^ mL^−1^.

### *Leishmania* transfection and selection of mutants

The LaLRR17 ORF was amplified and cloned in a modified phosphate-buffered saline (PBS) vector engineered to contain the sequence encoding the myc epitope and a tail of 6 histidines (a kind gift from Dr Carmen Fernandez-Becerra, Institute for Global Health, Barcelona, Spain). The LaLRR17 ORF in frame with the myc epitope and histidine tail was then excised and cloned into pXG1 (provided by Dr Steve Beverley, Washington University, St Louis, USA). DNA electroporation in *L. (L.) amazonensis* was performed as previously described (Kapler *et al*., 1990) using the construct pXG1 LaLRR17::myc or with the empty plasmid pXG1. Cells were then plated in medium 199–1% agar with G418 (20 mg mL^−1^). After 2 weeks, colonies were picked, expanded in liquid media and G418 concentrations were gradually increased to 160 mg mL^−1^.

### Production of LaLRR17 and GRP78 by *Escherichia coli*

A fragment of 2016 pb containing the coding sequence of LaLRR17 was cloned in the pAE vector (Ramos *et al*., 2004*a*) and used to transform *E. coli* BL21(DE3) pLysS. Bacteria were grown in 200 mL Luria-Bertani (LB) with 100 μg mL^−1^ ampicillin and expression was induced with 0.5 mm isopropyl-β-D-thiogalactopyranoside (IPTG) (Thermo Fisher Scientific, Waltham, Massachusetts, USA) for 4 h at 37°C. Bacteria were sedimented by centrifugation and suspended in 20 mL buffer A [100 mm Tris-HCl, pH 8.0, 12 mm sodium chloride (NaCl), 1 mm phenylmethylsulfonyl fluoride (PMSF)]. Cells were disrupted by sonication (Unique Ultrasonic DES500) and lysates were centrifuged. The pellet was suspended in 20 mL buffer B (100 mm Tris-HCl, pH 8.0, 500 mm NaCl, 8 m urea) and incubated at 4°C under rotation for 24 h. The lysate was again centrifuged, the pellet was discarded and 2 mL of the supernatant were added drop by drop to 10 mL buffer A (250 μL min^−1^). *β*-Mercaptoethanol was added to 5 mm and the sample was transferred to Ni-NTA column (Thermo Fisher Scientific). After washing the column with 20 mL buffer A, proteins were eluted with 50, 100 and 500 mm imidazole in buffer A and dialysed against PBS. GRP78 was produced using pQE10-GRP78 plasmid gently provided by Linda Hendershot, St. Jude's Children Hospital, USA, following a published protocol (Gaut and Hendershot, 1993). Protein concentration was determined using Bradford (BioRad, Hercules, California, USA), and lipopolysaccharide (LPS) content was calculated using *limulus amebocyte lysate* method (Pharma & Biotech Lonza, Bella Vista, Sidney, Australia), following the manufacturer's instructions.

### Isolation of bone marrow-derived macrophages (BMDMs)

BALB/c mice were euthanized and bone-marrow cells from femurs and tibias were collected in 10 mL R2030 medium (RPMI 1640 with 20% FCS and 30% supernatant from L929 cells). Cells were grown for 4 days, when 10 mL R2030 medium were added. After 3 days, cells were washed with PBS, detached with a cell scraper, counted and plated according to the experiment.

### Macrophage infection with *Leishmania*

Peritoneal macrophages were isolated as previously described (Velasquez *et al*., 2016). Peritoneal or BMDMs **(**8 × 10^5^ per well) in RPMI with (BMDM) or without (peritoneal macrophages) 10% FCS were transferred to 24-well plates covered with 13 mm circular coverslips. The choice of macrophage type depended on the number of cells required for each experiment, due to CEUA requests for reduction in the number of animals. After incubation at 37°C and 5% carbon dioxide (CO_2_) for 2 h (peritoneal cells) or overnight (BMDM), the medium was changed to RPMI with 10% FCS. When indicated, macrophages were pre-incubated for 1 h with anti-GRP78 1:150 (#3183, Cell Signaling). Infection was caused with *L. (L.) amazonensis* promastigotes at the beginning of the stationary phase (day 4) using a multiplicity of infection (MOI) of 5:1 in the presence or not of LaLRR17 (12.5, 25, 50 or 100 ng mL^−1^), LPS (2 ng mL^−1^, concentration found in 100 ng mL^−1^ LaLRR17) or GRP78 (4.6 μg mL^−1^). After removing non-internalized parasites by washing, cells were further incubated for 20 h. Cells were fixed with methanol, stained with InstantProv (NewProv, Pinhais, Paraná, Brazil) and mounted with Entellan (Merck, Darmstadt, Germany). One hundred macrophages were analysed per glass slide to determine the proportion of infected cells (IM) and amastigotes/infected macrophage, 3 coverslips were prepared for each condition, and 3 biological replicates (independent macrophages and *Leishmania* cultures) were performed for each experiment.

### Phagocytosis assay

For analysis of phagocytosis, 4 × 10^5^ BMDMs were transferred to 24-well plates covered with 13 mm circular coverslips. On the following day, cells were incubated with wild-type *L. (L.) amazonensis* in the presence of LaLRR17 or LPS using an MOI of 10. The plate was maintained on ice for 2 h and then incubated for 5, 30 or 60 min at 33°C and 5% CO_2_. Cells were then fixed with 4% paraformaldehyde for 10 min, washed with PBS and incubated with 50 mm of NH_4_Cl for 10 min, washed and blocked with 5% bovine serum albumin (BSA) in PBS for 2 h. Glass slides were incubated overnight with anti-*Leishmania* serum diluted 1:500 in PBS, washed with PBS and incubated for 1 h with a mix containing anti-mouse immunoglobulin G (IgG) (H + L) 488 Alexa fluor 1:1000, Phalloidin 568 Alexa fluor 1:100 and diamidino-2-phenylindole (DAPI) 1:1000. After 5 washing cycles, cells were mounted in ProLong (Thermo Fisher Scientific). For calculation of the phagocytosis index, 500 macrophages were analysed, and promastigotes were classified and quantified as attached (labelled in green and blue) or internalized (labelled only with blue by DAPI).

Anti-*Leishmania* serum was obtained by infecting BALB/c mice with *L. (L.) amazonensis*. Blood was collected around 10 weeks after infection; serum was obtained after centrifugation, and was stored in frozen aliquots.

### Sodium dodecyl sulphate-polyacrylamide gel electrophoresis (SDS-PAGE) and western blot

To obtain total protein extracts, parasites were washed twice with PBS, suspended at 10^9^ cells/300 μL in PBS + Proteoblock 1× (Fermentas, Waltham, Massachusetts, USA) and lysed by 8 freeze/thaw cycles (liquid nitrogen and 42°C). Soluble proteins were obtained after centrifugation at 12 000 ***g*** for 3 min and quantified by Bradford (BioRad).

SDS-PAGE (10% acrylamide:bisacrylamide separating gels) and western blots were performed as we previously described (Teixeira *et al*., 2015), using 10 μg of promastigote proteins and 1 μg of recombinant LaLRR17 or GRP78. Blocking was done overnight with PBS with 5% milk and 0.1% Tween 20, followed by incubation with primary antibody for 2 h and with secondary antibody for 1 h, both in PBS with 2.5% milk and 0.1% Tween 20. Three washing cycles (for 10 min) with 0.05% Tween 20 in PBS were performed after the incubations with primary and secondary antibodies. The following antibodies were used: anti-myc 1:3000 (Thermo Fisher Scientific) and anti-mouse horseradish peroxidase (HRP) 1:3000 (KPL Seracare, Milford, Massachusetts, USA) (for 1 h), anti-GRP78 ab32618 (Abcam, Cambridge, United Kingdom) 1:1000 and anti-rabbit HRP (Cell Signaling) 1:1000, anti-His 1:2500 (Thermo Fisher Scientific) and anti-mouse HRP 1:1000 (Sigma-Aldrich).

### Affinity chromatography

Affinity chromatography was performed using CNBr-activated Sepharose™ 4B (GE Healthcare, Chicago, Illinois, EUA), following the manufacturer's protocols. Briefly, 5 μg of recombinant LaLRR17 or BSA (control) were immobilized by incubation with the resin in 0.1 m NaHCO_3_ (pH 8.3), 0.5 m NaCl for 18 h at 4°C, and in 0.1 m Tris-HCl, pH 8.0 for 2 h at room temperature (RT). After sequential washing cycles with 0.1 m acetic acid, pH 4.0, with 0.5 m NaCl and with 0.1 m Tris-HCl (pH 8)–0.5 m NaCl (5 washing cycles with each solution), resins were incubated for 18 h at 4° C with 0.5 mg macrophage extract. This extract was prepared by lysis of cells in PBS with 1% NP40, centrifugation and recovery of the supernatant. After washing resins with PBS, bound proteins were sequentially eluted with 10% SDS in PBS, 8 m urea in PBS and 1 m NaCl.

### Mass spectrometry identification of LaLRR17-binding proteins

Eluted proteins from affinity chromatography were separated by SDS-PAGE and visualized by Coomassie blue G250 staining. Gel bands were extracted and transferred to microtubes. Trypsin digestion directly from the gel fragments (*in gel* digestion) and further processing for analysis by liquid chromatography coupled to sequential mass spectrometry (LC-MS/MS) in the nanoLC Easy-LTQ Orbitrap Velos-ETD equipment were performed in Centro de Facilidades para a Pesquisa, ICB-USP (CEFAP), according to CEFAP protocols. The proteins in the gel bands were identified by correlating the peptide masses in the MS/MS spectra with the protein sequences in the UniProt database.

### Immunofluorescence for GRP78

BMDMs were plated at 3 × 10^5^ cells per well over coverslips in 48-well plates for 24 h. Cells were then fixed by incubation with 4% paraformaldehyde (PFA) for 10 min, washed twice with PBS and incubated with 50 mm (NH_4_)Cl for 10 min. After 3 washing cycles with PBS and blocking with PBS containing 5% BSA for 2 h, coverslips were incubated for 2 h with anti-GRP78 ab32618 (Abcam) diluted 1:50 in PBS with 5% FCS.

Alternatively, BMDMs were washed twice with PBS and incubated for 30 min at RT with Fc Block (BD Biosciences, Franklin Lakes, New Jersey, USA) 1:100 in PBS with 5% FCS. Blocking solution was removed and cells were incubated with anti-GRP78 antibody for 30 min at RT. Wells were washed twice with PBS and cells were fixed by incubation with 4% PFA for 10 min. After 2 washing cycles with PBS, cells were incubated with 50 mm (NH_4_)Cl for 10 min.

For both protocols, coverslips were then washed with PBS and incubated for 30 min with anti-rabbit IgG (H + L) Alexa Fluor 488 (1:1000) and DAPI (1:1000) in PBS with 5% FCS. After washing cycles, coverslips were mounted in ProLong (Thermo Fisher Scientific). Images were acquired using a DMI6000B/AF6000 (Leica, Wetzlar, Germany) fluorescence microscope coupled to a digital camera system (DFC 365 FX) (Leica).

### Binding assay with LaLRR17 and GRP78

For analysis of binding of LaLRR17 to GRP78, 1 μg LaLRR17 or 1 μg BSA in 100 μL PBS were incubated in 96-well plates (Costar EIA/RIA high binding) for 18 h at 4°C. Wells were blocked with 150 μL PBS with 5% BSA for 2 h, washed 6 times with PBS and incubated with 4.6 μg mL^−1^ GRP78 for 18 h at 4°C. Wells were incubated with anti-GRP78 1:500 (Abcam ab32618) for 2 h, washed 6 times with PBS and incubated with anti-rabbit antibody HRP (Cell Signaling) diluted 1:1000 for 1 h. After 6 washing cycles with PBS, wells were incubated with TMB microwell peroxidase substrate (KPL Inc.) following the manufacturer's guidelines and read at 450 nm.

### Molecular docking

Molecular docking was used to test the binding affinity of LaLRR17 using a structure created by AlphaFold software, against the structures of GRP78 (AlphaFoldDB-P20029), GRP75 (AlphaFoldDB-P38647) and HSP-71 (AlphaFoldDB-AF-P63017-F1). The solvated docking software HADDOCK was used to model LaLRR1 regions against the solved structure of GRP78, GRP75 and HSP-71; the easy interface was used as there are no constraints set. PRODIGY software was used to predict the binding affinity for each region of the LaLRR17 to GRP78.

### Statistical analysis

GraphPad software (San Diego, CA, USA) was used to perform all analyses. We employed one-way analysis of variance (ANOVA) followed by Tukey's multiple comparison test (for 3 or more samples), or *t*-test (for comparison of 2 conditions).

## Results

### Identification of *L. (L.) amazonensis* LaLRR17

**Figure 1.**
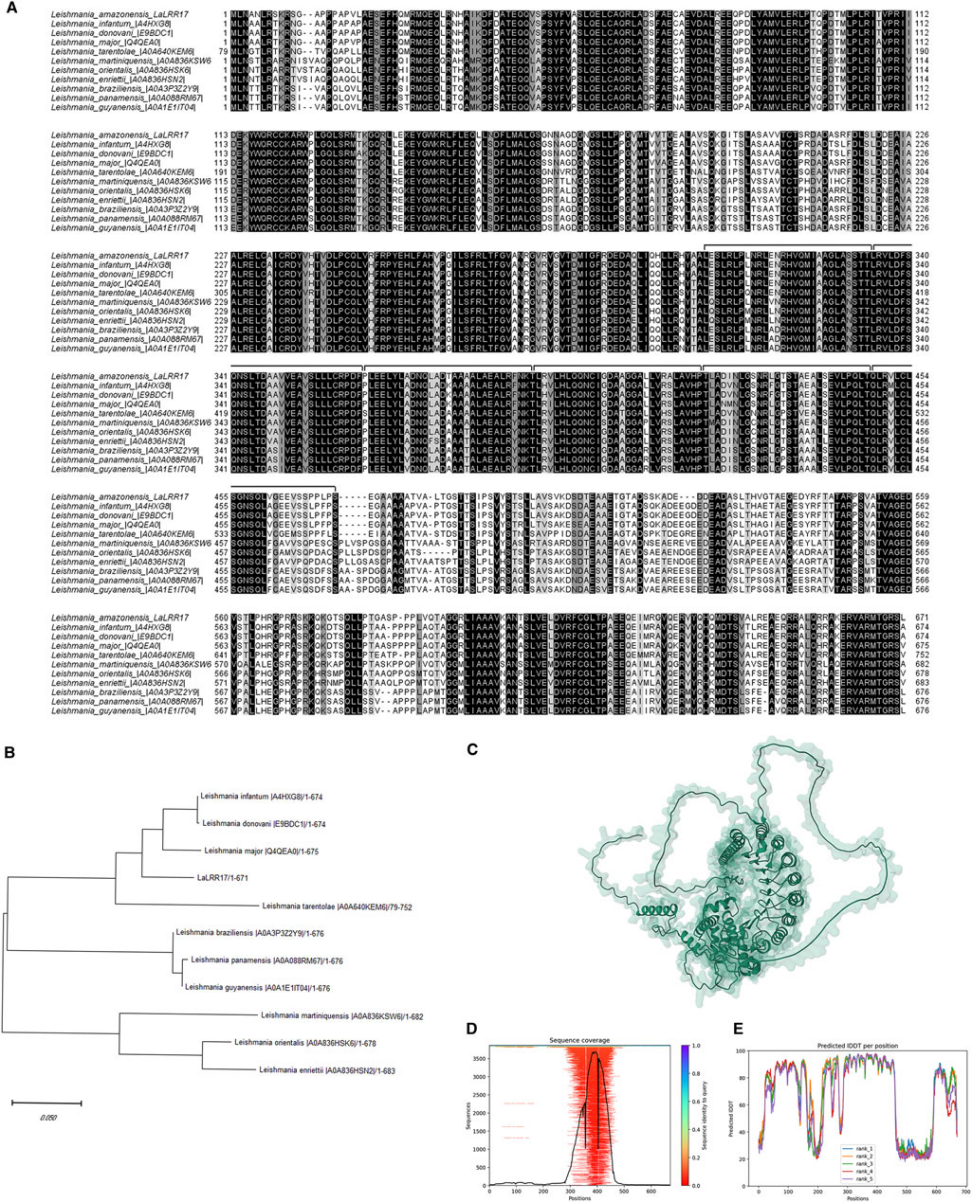
LRR17 protein sequence in *Leishmania*. (A) Multiple alignment of the *Leishmania (L.) amazonensis* LRR17 translated sequence (GenBank EU906911.1) with sequences from several *Leishmania* species performed using MUSCLE. Identical amino acids in all sequences are shaded in black; amino acids conserved in 50% of the sequences are shaded in grey. Black lines indicate the LRR units. (B) Phylogenetic tree constructed with protein sequences using the ML method. Bar scale indicates 5% amino acid divergence. (C) Model of LaLRR17 structure predicted by the server AlphaFold. (D) Sequence coverage plot showing the number of homologues identified across the representative sequence and coloured by the sequence identity of the homologues. (E) A plot of the pLDDT score per position for each of the 5 AlphaFold models predicted.

The characterization and sequencing of a segment of chromosome 17 of *L. (L.) amazonensis* containing the *META1* and *META2* genes (Uliana *et al*., 1999; Ramos *et al*., 2011) led to the identification of a neighbouring ORF encoding a protein containing 6 region LRRs that was named LaLRR17 (Fig. 1). The 5′ and 3′ untranslated region (UTR) regions were mapped through RACE, allowing determination of the complete ORF (original sequence deposited in GenBank under EU906911) (Ramos *et al*., 2004*b*). The predicted polypeptide sequence contains no signal peptide or glycosylphosphatidylinositol (GPI)-anchoring sites. Analysis of the LaLRR17 peptide sequence (https://www.ebi.ac.uk/interpro/) (Mitchell *et al*., 2019) identified a region with homology with the superfamily of LRR domains (IPR032675). The structure predicted by the server AlphaFold indicates that the LRR motifs (with 28–30 amino acids in length) are mostly located in the central region of the protein (Fig. 1C).

Similar ORFs were identified in several other *Leishmania* species by searching published genome data (Fig. 1A). UniProt Knowledge base (UniProtKB) identified homologues in *Leishmania major*, *Leishmania infantum* and *Leishmania braziliensis*, identified as putative hypothetical proteins, sharing 92, 92 and 78% identity, respectively, with LaLRR17. Interestingly, in a phylogenetic analysis using the maximum-likelihood (ML) method, LRR17 sequences from *Viannia* subgenus cluster separately from *Leishmania* and *Sauroleishmania* subgenera branch (Fig. 1B).

### Characterization of *L. (L.) amazonensis* line overexpressing LaLRR17

**Figure 2.**
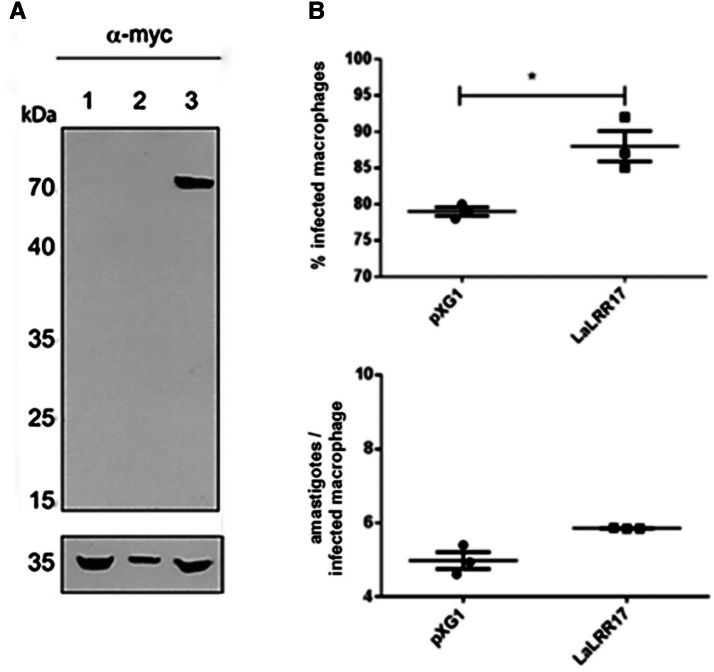
Infection of macrophages with transgenic *L. (L.) amazonensis* overexpressing LaLRR17-myc and control lines. (A) Immunoblotting of total protein extracts from *L. (L.) amazonensis* log phase promastigotes probed with a monoclonal anti-myc antibody. Equal numbers of parasites (2 × 10^7^) were loaded per track: (1) wild-type line; (2) *L. (L.) amazonensis* (pXG1 *NEO*) and (3) *L. (L.) amazonensis* (pXG1 *NEO* LaLRR17). The bottom panels show the same blot incubated with an anti-glyceraldehyde-3-phosphate dehydrogenase (GAPDH) as a loading control. (B) Peritoneal macrophages from BALB/c mice were infected with *L. (L) amazonensis* (pXG1 NEO LaLRR17::myc/His) and (pXG1 NEO) for 24 h at an MOI of 5. Percentage of infected cells (top graph) and numbers of amastigotes per infected macrophage (bottom graph); *t*-test, *P ⩽ 0.05. A representative experiment of 3 with similar profiles is shown.

To gather functional information on LaLRR17 protein, we produced a transgenic strain overexpressing the protein, named (pXG1 NEO LaLRR17::myc/His). *Western blot* confirmed the expression of exogenous myc-tagged LaLRR17 in the transgenic overexpressor (pXG1 NEO LaLRR17::myc/His), but not in the transgenic control (pXG1 NEO) promastigotes (Fig. 2A). These transgenic parasites were then used for infection of peritoneal macrophages from BALB/c mice for 24 h, to analyse if LRR17 affects parasite entry and/or survival in the initial phase, before parasite multiplication. Significantly higher percentages of infected cells (*P* ⩽ 0.05) were observed for LaLRR17 overexpressing parasites, and a non-significant increase in the number of parasites per macrophage was also observed for this line (Fig. 2B). Independent clones selected after transfection exhibited the same phenotype (data not shown). These results suggest that LaLRR17 contributed to macrophage infection.

### Soluble LaLRR17 increases phagocytosis of *L. (L.) amazonensis*

**Figure 3.**
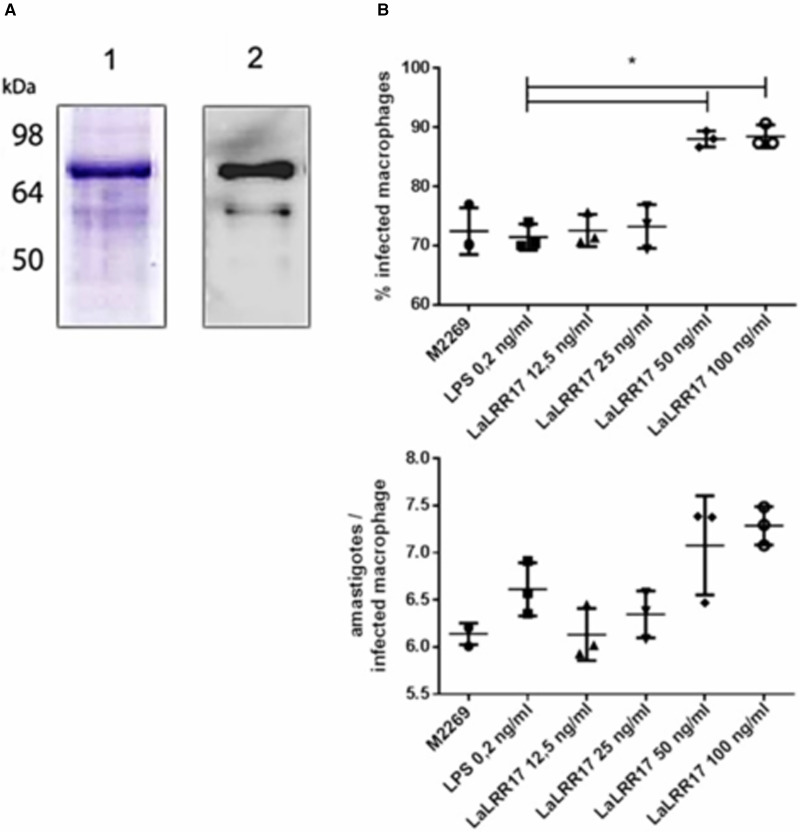
Production of recombinant LaLRR17 and its effect on macrophage infection by *L. (L.) amazonensis.* (A) SDS-PAGE (1) and western blot with anti-His 1:2500 (2) showing recombinant LaLRR17 purified from bacteria. (B) Peritoneal macrophages from BALB/c mice were infected with wild-type *L. (L) amazonensis* for 24 h at an MOI of 5 in the presence of LPS or 12.5, 25, 50 or 100 ng mL^−1^ of LaLRR17. Percentage of infected cells (top graph) and numbers of amastigotes per infected macrophage (bottom graph). ANOVA followed by post-test of Tukey's, *P ⩽ 0.05. Means and standard deviations of 3 independent experiments.

As previously mentioned, LaLRR17 has no domains for membrane anchoring or secretion. However, many virulence factors of *Leishmani*a are present in extracellular space, after secretion by non-classical pathways. To test whether the soluble protein would affect infection, we produced the recombinant His-tagged protein in the BL21(DE3) pLysS system using the pAE plasmid LaLRR17 (Ramos *et al*., 2004*a*). The recombinant LaLRR17 protein, which was not soluble after induction, was obtained from 8 m urea pellet and refolded by drop dilution (Fig. 3A, SDS-PAGE and western blot). Soluble LaLRR17 was then used in macrophage infections for 24 h with wild-type *L. (L.) amazonensis*.

Since LaLRR17 produced in bacterial systems is expected to contain small traces of LPS, which may activate macrophages and reduce infection (Meng and Lowell, 1997), we added LPS at the highest concentration found in our LaLRR17 preparations (2 EU mL^−1^, corresponding to 0.2–0.4 ng endotoxin mL^−1^) as the control condition. Different concentrations of LaLRR17 were assayed in macrophage infections for 24 h (4 h of contact with the parasite and 20 h of incubation at 34°C), and a significant difference (*P* ⩽ 0.05) in the proportion of infected cells was observed in the presence of 50 and 100 ng mL^−1^ of LaLRR17, but not in lower concentrations (Fig. 3B). A non-significant increase was noted in the number of parasites per infected macrophage in the presence of LaLRR17 (Fig. 3B). LPS did not affect any of the parameters analysed.

The results obtained with the recombinant protein were similar to those observed with the parasites overexpressing LaLRR17, indicating that the recombinant protein is active. Besides, these results suggest that if LaLRR17 is present in extracellular space, shed by parasites or made available after neighbouring parasites death, it probably exerts an effect on promastigote infection.

**Figure 4.**
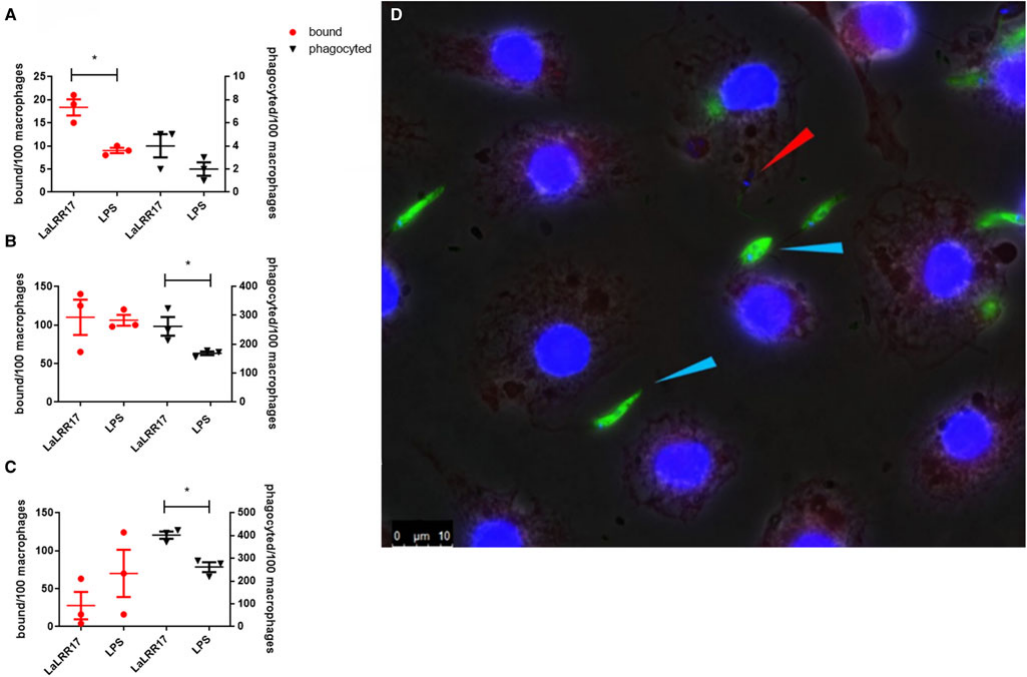
Effect of LaLRR17 on binding and on phagocytosis of *L. (L.) amazonensis* by BMDM. Each graph shows promastigotes bound (circles in red, left) and phagocytosed (triangles, right) by 100 BMDM (MOI of 10) in the continuous presence of 100 ng mL^−1^ LaLRR17 after (A) 5 min, (B) 30 min and (C) 60 min of contact between parasite and macrophage at 37°C. Experiment representative of 3 (A) or 2 (B, C) independent experiments; *t*-test, *P* ⩽ 0.05.

We then analysed whether the increase observed in infection experiments (24 h) was due to an action of LRR17 in the initial stages of phagocytosis. To test this hypothesis, we performed a phagocytosis assay, in which parasites contact macrophages for very short periods. Promastigotes were incubated with macrophages (BMDM) in the presence or absence of LaLRR17 for 5, 30 or 60 min. Promastigotes adhered to the host cell, but not internalized, were visualized by fluorescence microscopy on non-permeabilized cells stained in green and blue (anti-*Leishmania* + secondary antibody Alexa 488 and DAPI, respectively), while internalized parasites were labelled only in blue (DAPI) (Fig. 4D). Phagocytosis of *Leishmania* was assessed quantitatively in terms of the number of promastigotes internalized per 100 macrophages, and we determined the number of parasites bound to these cells. Figure 4A–C shows 1 graph for each time point, and each graph shows bound and internalized parasites. Interestingly, LaLRR17 led to an increase in bound promastigotes after 5 min of macrophage–parasite contact, and in internalized promastigotes after 30 and 60 min contact, indicating that the protein affects the binding and further phagocytosis of *Leishmania*, probably by interaction with a ligand on the macrophage surface.

### Identification of GRP78 as a ligand for LaLRR17 by affinity chromatography followed by mass spectrometry

**Figure 5.**
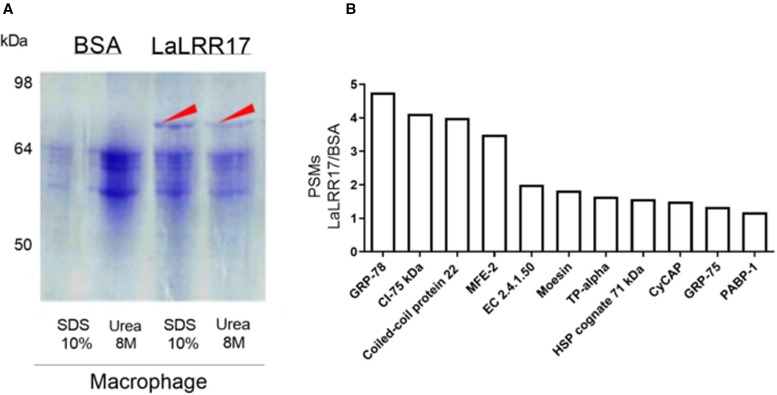
Identification of macrophage proteins that bind to LaLRR17. (A) SDS-PAGE 10% showing proteins eluted (with SDS or urea) from BSA and LaLRR17 columns. The arrow points to the differential band recovered from the gel and analysed in (B). (B) Proteins with PSM ratios above 1 for LaLRR17/BSA.

In order to identify macrophage proteins that bind to LaLRR17 and can mediate *Leishmania* phagocytosis, we employed affinity chromatography with immobilized LaLRR17. The recombinant protein was coupled to CNBr-activated Sepharose™ 4B beads and incubated with soluble lysates of BMDM from BALB/c mice. BSA was coupled to the resin as a control and submitted to the same protocol. The eluates of the 2 columns were analysed on SDS-PAGE, and the differential band between 64 and 98 kDa retained in LaLRR17 but not on BSA column (Fig. 5A) was collected and submitted to mass spectrometry analysis (Supplementary Table 1). A region of the same size on the BSA lane was also collected and analysed as negative control. Peptide–spectrum matches (PSMs) were used to derive the relative abundance of each protein in LaLRR17 and BSA eluates, as previously described (Shteynberg *et al*., 2013). High PSM ratios for LaLRR17/BSA indicate potential affinity for LaLRR17. Among the identified proteins, 11 had ratios above 1, 5 of which were equal or above 2 and 4 of which were above 3 (Fig. 5B). Supplementary Table 1 lists the PSMs of all proteins identified in the affinity chromatography.

The GRP78, also known as binding immunoglobulin protein or heat shock 70 kDa protein 5, was the protein with the highest PSMs for LaLRR17 relative to BSA (Fig. 5B), suggesting its ability to bind to LaLRR17. However, other proteins similar to GRP78 also bound to LaLRR17, such as GRP75 and HSP cognate 71 kDa, which have 46.8 and 60.8% amino acid identity with GRP78, respectively (Supplementary Fig. 1).

### Prediction of binding between LaLRR17 and GRP78, GRP75 and HSP-71

The docking of LaLRR17 and GRP78 proteins, whose structure was determined using AlphaFold, was performed using HADDOCK software (Ibrahim *et al*., 2020) setting no restrictions and therefore using the easy interface (Elfiky and Ibrahim, 2021). To define the surface residues of the LaLRR17 protein as active and passive, we employed the automatic definition mode. HADDOCK software is widely employed for protein docking, to investigate the molecular interactions between a target protein and its partner molecules such as ligands, substrates or other proteins. It is based on constraints and experimental information. It employs an integrative approach that combines structural and experimental data, such as nuclear magnetic resonance and mass spectrometry, to enhance the accuracy of the results.

**Figure 6.**
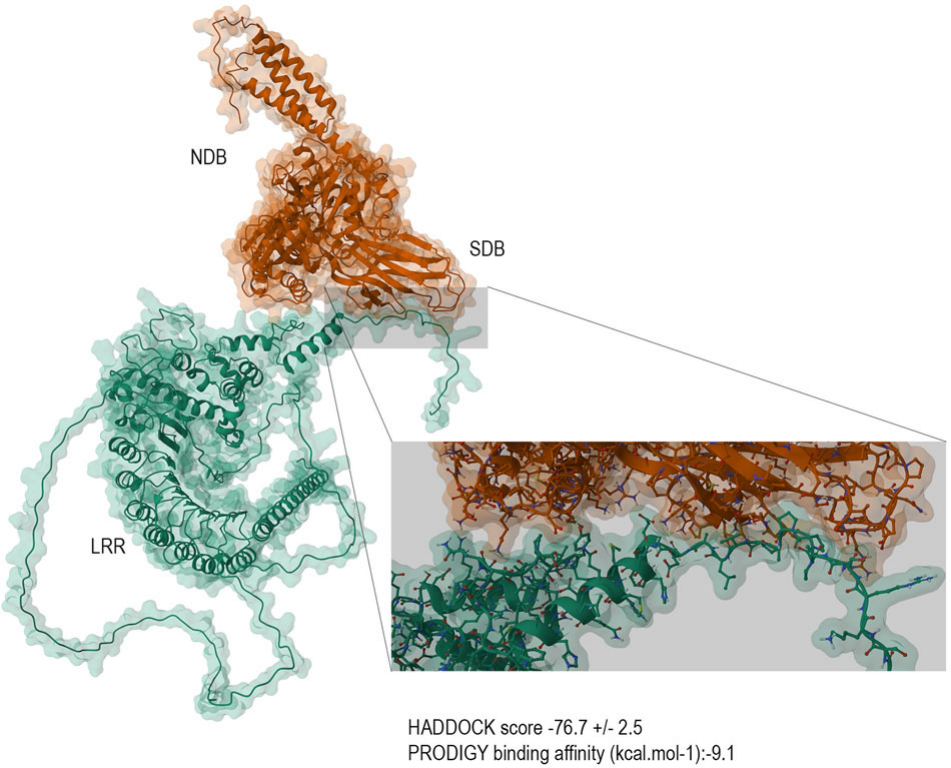
Prediction of binding between LaLRR17 and GRP78. Images show the binding for each of the docking assays (the best-formed complexes from each docking experiment). Orange drawing represents GRP78, indicating SBD and nucleotide-binding domain (NBD) regions, and the green drawing represents LaLRR17, indicating the LRR region.

For GRP78, we used the active residues characterized in previous studies (Almagro Armenteros *et al*., 2017) and adapted to the sequence of mouse GRP78 as follows: I427, T429, V430, V433, T435, F452, S453, V462 and I464 (Fig. 6A). The best complex created by HADDOCK software achieved the score of −76.7 ± 9.1, with the predicted binding site of the LaLRR17 protein to GRP78 in good agreement with studies that have identified the binding domain of pathogens to membrane GRP78, such as proteins from Zika virus and severe acute respiratory syndrome coronavirus 2 (Ibrahim *et al*., 2020; Elfiky and Ibrahim, 2021).

We have also analysed the docking of LaLRR17 and GRP75 or HSP-71, the 2 other chaperones similar to GRP78 already mentioned. The results of the predictions are shown in Supplementary Fig. 2. All chaperones bind, on average, to the first 25 amino acids of the N-terminal region of LaLRR17. The interaction with GRP78 occurs in the substrate binding domain (SBD) region, which usually binds to peptides/proteins.

**Figure 7.**
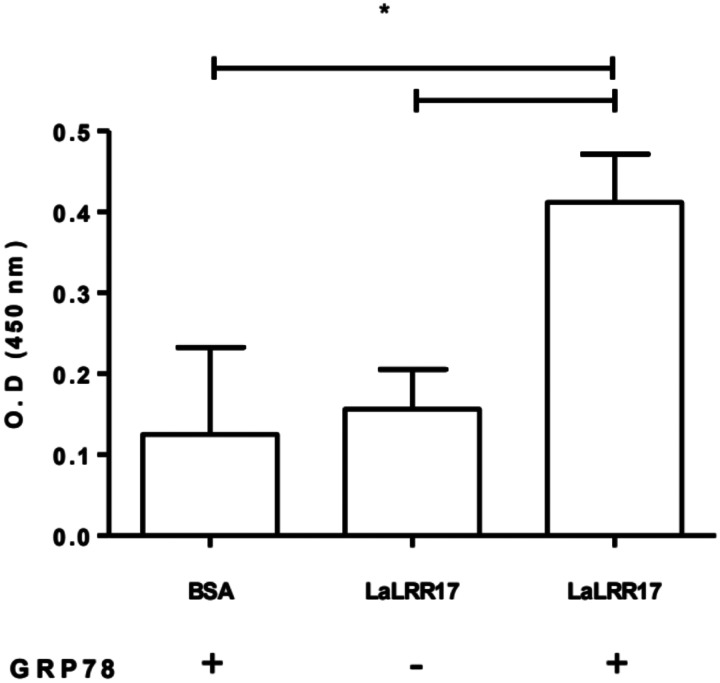
Binding of recombinant GRP78 to LaLRR17. Binding of GRP78 to 1 μg LaLRR17 or BSA estimated by enzyme-linked immunoassay using anti-GRP78 (ab32618, Abcam) diluted 1:500. Results representative of 3 independent experiments, statistical analysis used: ANOVA and post-test of Tukey's, *P* ⩽ 0.05.

To confirm that GRP78 interacts with LaLRR17, we produced a recombinant GRP78 and performed a binding assay using both proteins. The results shown in Fig. 7 indicate that GRP78 exhibited significantly higher binding to LaLRR17 than to BSA, indicating specific binding between the 2 proteins. Unfortunately, biophysical approaches could not be performed to validate these observations due to the low concentrations of LaLRR17 always obtained after induction and refolding.

**Figure 8.**
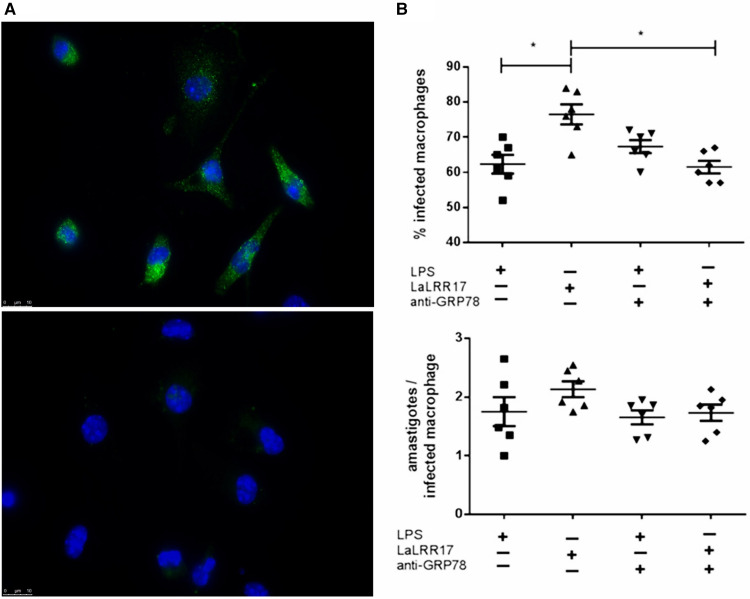
Analysis of GRP78 expression and effects on macrophage infection. (A) Immunofluorescence showing GRP78 labelling in live BMD macrophages (top image, anti-GRP78 and anti-rabbit 488 Alexa fluor) and no labelling with secondary antibody only (bottom image, anti-rabbit 488 Alexa fluor). (B) Macrophage infection in the presence or absence of LaLRR17 and anti-GRP78. Peritoneal BALB/c macrophages pre-incubated or not with anti-GRP78 were infected at an MOI of 5 with *L. (L.) amazonensis* in the presence or absence of 100 ng mL^−1^ of LaLRR17, for 24 h. Percentage of infected cells (top graph) and numbers of amastigotes per infected macrophage (bottom graph). ANOVA followed by post-test of Tukey's, *P* ⩽ 0.05. Results of 1 experiment representative of 3.

Since the literature is scarce on data about the expression of GRP78 by macrophages, we examined whether it was present on the macrophage surface. Positive staining with anti-GRP78 of non-permeabilized cells suggested the presence of GRP78 on the cell surface (Fig. 8A).

We then evaluated its participation in the infection enhancement promoted by LaLRR17. Blocking GRP78 with a specific antibody did not affect infection percentage in macrophages in the absence of soluble LRR17 (Fig. 8B). However, the increased infection observed in the presence of soluble LRR17 was reversed by the incubation with anti-GRP (Fig. 8B), reinforcing that the GRP78 protein on the macrophage surface mediated the effect of LaLRR17.

## Discussion

LRR motifs present in several proteins of mammals and plants are involved in protein–protein interactions (Kobe and Kajava, 2001). In this paper, we described the characterization of a *Leishmania-*conserved gene encoding LaLRR17, a leucine-rich protein, found in the vicinity of the *META* genes in chromosome 17. The study of protein abundance and cellular localization proved challenging since, despite several attempts to immunize mice or rabbits with recombinant proteins or with synthetic peptides, we could not generate antibodies that specifically detect LaLRR17 (data not shown).

Similar to what has been suggested for PPG, PSA-2 and Inl-A-like protein, we hypothesized that LaLRR17 may interact with macrophages. The LaLRR17 protein does not have any membrane-anchoring domain and is predicted to be soluble and localized in the cytosol (DeepLoc-1.0 value of 0.57, corresponding to soluble cytoplasm) (Almagro Armenteros *et al*., 2017). It does not exhibit an N-terminal secretion signal peptide and therefore would not be secreted by the classical eukaryotic secretion pathway. Analyses of *Leishmania* secretomes show that only a few proteins in the extracellular environment do possess classical secretion signals (Silverman *et al*., 2008; Pissarra *et al*., 2022), with most proteins being secreted through extracellular vesicles (EVs). EVs released by *Leishmania* contribute to the immunomodulation of the host and consequently to the establishment of infection (Corrales *et al*., 2010; Barbosa *et al*., 2018; Garg *et al*., 2019; Rodriguez-Vega *et al*., 2021). In addition to secretion by EVs, LaLRR17 might be shed by a portion of promastigotes present in the initial inoculum that initiate programmed cell death in the mammalian host (El-Hani *et al*., 2012), releasing cytosolic proteins to the extracellular environment (Santarem *et al*., 2007).

We have shown that parasites overexpressing LaLRR17 infected a higher percentage of macrophages than the wild-type strain. Similarly, the presence of the soluble recombinant LaLRR17 protein increased the proportion of infected cells. Moreover, soluble LaLRR17 led to an increase in the number of promastigotes bound to macrophages after incubations for only 5 min, and in the number of internalized parasites after 30 min or longer. These data indicated that LaLRR17 and its ligand in the macrophage enhance parasite binding and phagocytosis. The best-studied receptors related to *Leishmania* phagocytosis are complement receptors 3 (CR3) and 1 (CR1), mannose receptors, Fc gamma receptor (mainly FcyRII-B2) and fibronectin receptors (Guy and Belosevic, 1993; Podinovskaia and Descoteaux, 2015; Podinovskaia and Russell, 2015). As previously mentioned, the ER also contributes to phagocytosis, as attested by the presence of proteins such as calnexin and calreticulin on the cell surface (Muller-Taubenberger *et al*., 2001). These 2 proteins appear to participate in *Leishmania* phagocytosis, as well as parasite GP63, which contains a Glc_1_Man_6_ GlcNac_2_ structure recognized by calnexin and calreticulin (Olafson *et al*., 1990; Garin *et al*., 2001). None of these macrophage proteins, however, has been shown to interact with *Leishmania* proteins containing LRRs.

Affinity chromatography followed by mass spectrometry identified chaperones from the HSP-70 family including GRP78, GRP75 and HSP cognate 71 kDa as ligands for LaLRR17, with GRP78 being the most abundant. GRP78 is the most well-studied protein in the GRP family (Wang *et al*., 2009; Behnke *et al*., 2015; Pobre *et al*., 2019). Due to the presence of the KDEL (Lys–Asp–Glu–Leu) peptide for retention in the ER, most GRP78 is localized within this organelle, but in some circumstances, it can be redistributed to the cytosol, nucleus, mitochondria and plasma membrane, and can even be secreted (Suzuki *et al*., 1991). A determinant factor for change in GRP78 cellular localization may be ER stress, when proteins that reside inside the ER can translocate to the cytosol, cell membrane or extracellular space (Pelham, 1990), contributing to various cellular pathologies (Arap *et al*., 2004; Tsai *et al*., 2015; Wiersma *et al*., 2015; Ha *et al*., 2020). On the cell surface GRP78 functions as a signalling molecule and may play an important role in regulating pro-proliferative/anti-apoptotic, pro-migratory, signalling pathways and pathogen entry (Gonzalez-Gronow *et al*., 2009; Ibrahim *et al*., 2019; Lenin *et al*., 2019).

The mechanism by which *L. (L.) amazonensis* may induce ER stress is not well understood, but mild ER stress response induced by *Leishmania* infection may represent a common pathogenic mechanism among different *Leishmania* species and may be part of the strategies to survive in the host cell (Galluzzi *et al*., 2016).

Some reports have described GRP78 on the surface of mouse macrophages (Misra *et al*., 2005; Lu *et al*., 2010) and its participation in signalling pathways triggered by *α*2-macroglobulin binding, leading to macrophage activation and chemotaxis (Misra and Pizzo, 2008). We confirmed here that murine-resident peritoneal macrophages express GRP78 on their surface and that blocking this protein using a specific antibody prevented the increase in infection induced by LaLRR17. Interestingly, blocking GRP78 did not reduce infection in the absence of exogenous LaLRR17. We believe LRR17 expression is low in wild-type parasites and its presence is even lower under the conditions used in infection experiments: day 4 culture promastigotes, centrifuged and resuspended in medium, added to macrophages. Under such conditions, we do not expect to have considerable amounts of soluble endogenous LaLRR17. On the contrary, during the transmission of promastigotes to mammals, a part of the parasites in the inoculum die and release LRR17 in the lesion environment. Such a condition may be mimicked by *in vitro* infections in the presence of exogenous recombinant LRR17, in which LRR17 augments infection by binding to GRP78, and thus the blocking of GRP78 precludes this increase.

These findings suggest that LaLRR17 interacts with macrophage GRP78 and increases parasite binding and phagocytosis, enhancing infection. Interestingly, GRP78 is used as a receptor that mediates the binding of the fungus *Rhizopus oryzae* to its host cell (Liu *et al*., 2010) and its endocytosis (Gebremariam *et al*., 2014). In infections with Zika virus, the envelope protein is responsible for binding, entry and cell fusion, and one of the receptors responsible for virus endocytosis is GRP78 (Royle *et al*., 2020; Khongwichit *et al*., 2021). In *Coxsackievirus* A9, the virus invades the cell by binding to GRP78 and integrin *α*v*β*3 (Triantafilou *et al*., 2002), as well as to the ACE2 protein (Ibrahim *et al*., 2020; Carlos *et al*., 2021).

This study described for the first time the LaLRR17 protein as a possible virulence factor of *Leishmania* and its interaction with the principal ER chaperone and cell surface co-receptor, GRP78. Soluble LaLRR17 may be available after secretion by the parasite or parasite death and will increase phagocytosis of promastigotes. Although the lack of an anti-LRR17 antibody precluded precise analysis of LRR17 location and secretion, our findings suggest functions for this protein in parasite–host interaction. This is the first report of the involvement of GRP78 in a parasitic infection.

## Supplementary material

For supplementary material accompanying this paper visit https://doi.org/10.1017/S0031182023000720. click here to view supplementary material
