# Supplementary material for: Leishmania (L.) amazonensis LaLRR17 increases parasite entry in macrophage by a mechanism dependent on GRP78
Source: Parasitology. 2023 Aug 9;150(10):922–33. doi: 10.1017/S0031182023000720 (PMC10577668; doi:10.1017/S0031182023000720)
Supplement: Supplementary file 1 [file S0031182023000720sup.zip › S0031182023000720sup003.docx]

| ID | Protein | Function | PSMs |
| --- | --- | --- | --- |
| P20029 | GRP-78 | Endoplasmic reticulum chaperone that plays a key role in protein folding and quality control in the endoplasmic reticulum lumen | 195 |
| Q91VD9 | CI-75kDa | Core subunit of the mitochondrial membrane respiratory chain NADH dehydrogenase (Complex I) that is believed to belong to the minimal assembly required for catalysis | 33 |
| P29341 | PABP-1 | Binds the poly(A) tail of mRNA, including that of its own transcript, and regulates processes of mRNA metabolism such as pre-mRNA splicing and mRNA stability | 26 |
| Q8BMS1 | TP-alpha | Mitochondrial trifunctional enzyme catalyzes the last three of the four reactions of the mitochondrial beta-oxidation pathway | 23 |
| P63017 | HSP cognate 71kDa | Protection of proteins from stress, folding and transport of newly synthesized polypeptides, activation of proteolysis of misfolded proteins, formation and dissociation of protein complexes | 22 |
| P26041 | Moesin | Ezrin-radixin-moesin (ERM) family protein that connects the actin cytoskeleton to the plasma membrane and thereby regulates the structure and function of specific domains of the cell cortex | 11 |
| P51660 | MFE-2 | Bifunctional enzyme acting on the peroxisomal beta-oxidation pathway for fatty acids | 7 |
| Q8K297 | EC 2.4.1.50 | Beta-galactosyltransferase that transfers beta-galactose to hydroxylysine residues of type I collagen | 6 |
| Q9JIG7 | Coiled-coil protein 22 | Involved in regulation of NF-kappa-B signaling | 4 |
| P38647 | GRP-75 | Chaperone protein which plays an important role in mitochondrial iron-sulfur cluster (ISC) biogenesis | 3 |
| Q07797 | CyCAP | Promotes integrin-mediated cell adhesion. May stimulate host defense against viruses and tumor cells | 3 |

Supplementary table 1: Functions of proteins with Peptide-Spectrum Matches (PSMs) ratios above 1 for LaLRR17/ BSA Data obtained by UNIPROT.
