# Supplementary material for: Leishmania (L.) amazonensis LaLRR17 increases parasite entry in macrophage by a mechanism dependent on GRP78
Source: Parasitology. 2023 Aug 9;150(10):922–33. doi: 10.1017/S0031182023000720 (PMC10577668; doi:10.1017/S0031182023000720)
Supplement: Supplementary file 1 [file S0031182023000720sup.zip › S0031182023000720sup004.docx]

| LaLRR17/GRP78 |  |
| --- | --- |
| [+] Parsed structure file LaLRR17/GRP78 | (2 chains, 1326 residues) |
| [+] No. of intermolecular contacts | 61 |
| [+] No. of charged-charged contacts | 6 |
| [+] No. of charged-polar contacts | 7 |
| [+] No. of charged-apolar contacts | 13 |
| [+] No. of polar-polar contacts | 0 |
| [+] No. of apolar-polar contacts | 14 |
| [+] No. of apolar-apolar contacts | 21 |
| [+] Percentage of apolar NIS residues | 39.39 |
| [+] Percentage of charged NIS residues | 32.54 |
| [++] Predicted binding affinity (kcal.mol-1) | -9.1 |
| [++] Predicted dissociation constant (M) at 34.0˚C | 3.1e-07 |
|  |  |
| LaLRR17/GRP75 |  |
| [+] Parsed structure file LaLRR17/GRP75 | (2 chains, 1350 residues) |
| [+] No. of intermolecular contacts | 80 |
| [+] No. of charged-charged contacts | 7 |
| [+] No. of charged-polar contacts | 19 |
| [+] No. of charged-apolar contacts | 21 |
| [+] No. of polar-polar contacts | 1 |
| [+] No. of apolar-polar contacts | 19 |
| [+] No. of apolar-apolar contacts | 13 |
| [+] Percentage of apolar NIS residues | 41.04 |
| [+] Percentage of charged NIS residues | 30.22 |
| [++] Predicted binding affinity (kcal.mol-1) | -11.0 |
| [++] Predicted dissociation constant (M) at 34.0˚C | 1.5e-08 |
|  |  |
| LaLRR17/HSP71kDa |  |
| [+] Parsed structure file LaLRR17/HSP71kDa | (2 chains, 1317 residues) |
| [+] No. of intermolecular contacts | 89 |
| [+] No. of charged-charged contacts | 6 |
| [+] No. of charged-polar contacts | 14 |
| [+] No. of charged-apolar contacts | 25 |
| [+] No. of polar-polar contacts | 1 |
| [+] No. of apolar-polar contacts | 31 |
| [+] No. of apolar-apolar contacts | 12 |
| [+] Percentage of apolar NIS residues | 40.12 |
| [+] Percentage of charged NIS residues | 30.42 |
| [++] Predicted binding affinity (kcal.mol-1) | -141 |
| [++] Predicted dissociation constant (M) at 34.0˚C | 8.4e-11 |
