# Supplementary figures and images for: Leishmania (L.) amazonensis LaLRR17 increases parasite entry in macrophage by a mechanism dependent on GRP78
Source: Parasitology. 2023 Aug 9;150(10):922–33. doi: 10.1017/S0031182023000720 (PMC10577668; doi:10.1017/S0031182023000720)

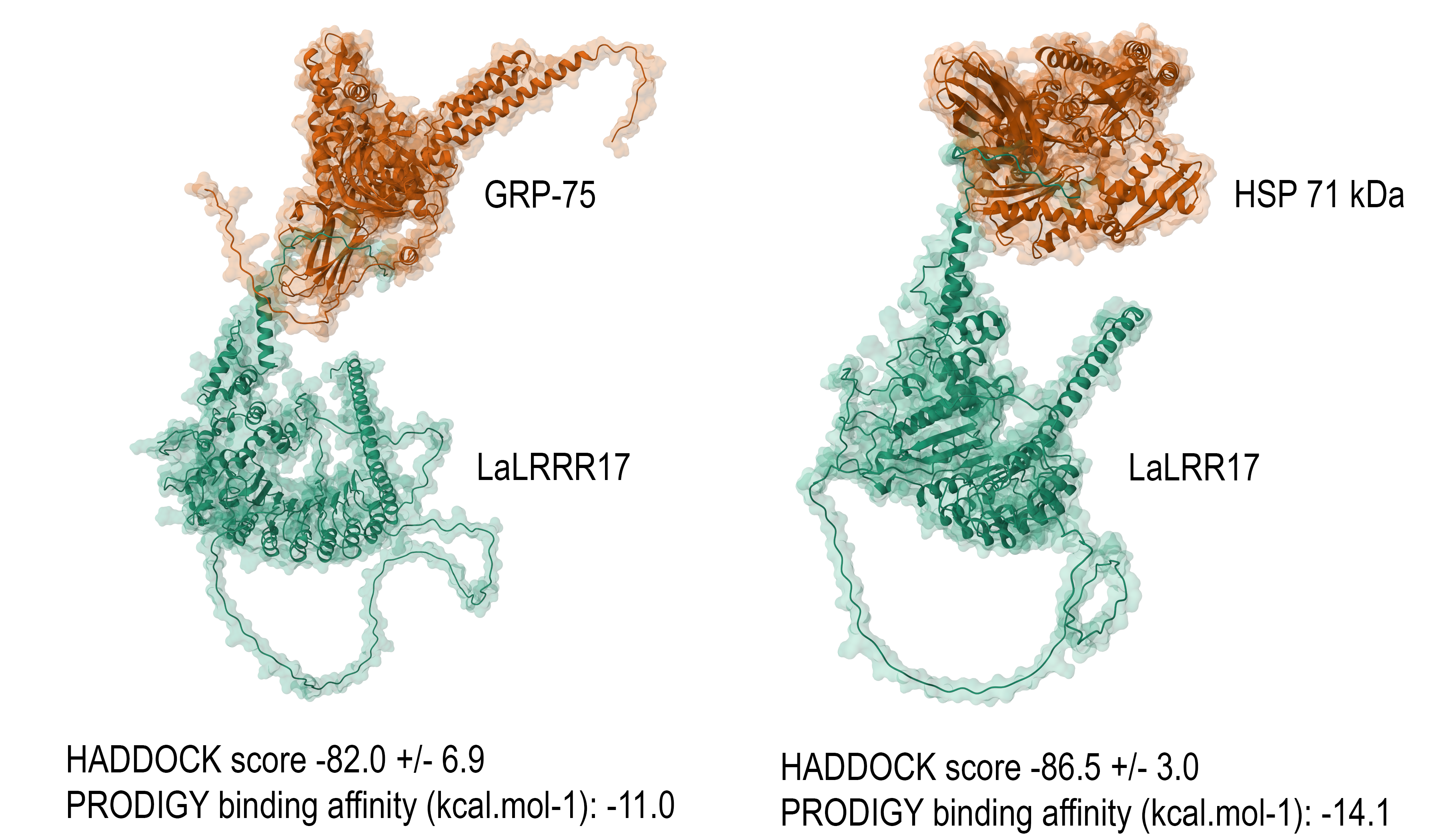

Supplement: Supplementary file 1 [file S0031182023000720sup.zip › S0031182023000720sup002.tif]

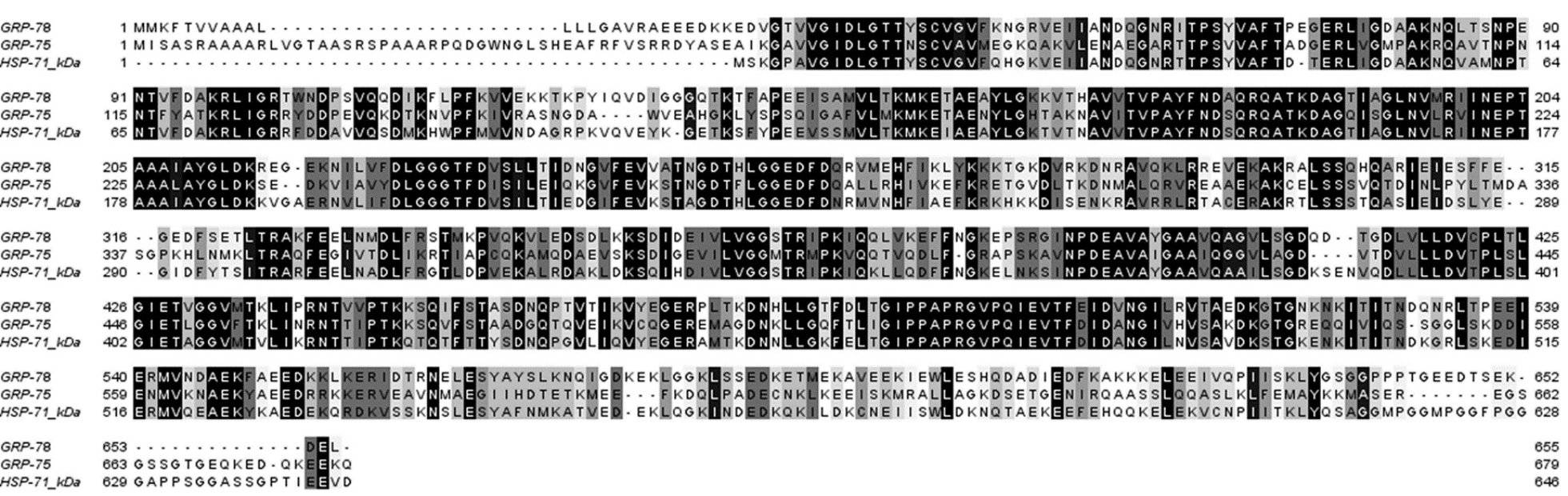

Supplement: Supplementary file 1 [file S0031182023000720sup.zip › S0031182023000720sup001.tiff]
